# Manual therapies for cervicogenic headache: a systematic review

**DOI:** 10.1007/s10194-012-0436-7

**Published:** 2012-03-30

**Authors:** Aleksander Chaibi, Michael Bjørn Russell

**Affiliations:** 1 Head and Neck Research Group, Research Centre, Akershus University Hospital, 1478 Lørenskog, Oslo Norway; 2 Institute of Clinical Medicine, Akershus University Hospital, University of Oslo, 1474 Nordbyhagen, Norway

**Keywords:** Randomized clinical trials, Manual therapies, Physiotherapy, Chiropractic, Cervicogenic headache, Treatment

## Abstract

This paper systematically reviewed randomized clinical trials (RCT) assessing the efficacy of manual therapies for cervicogenic headache (CEH). A total of seven RCTs were identified, i.e. one study applied physiotherapy ± temporomadibular mobilization techniques and six studies applied cervical spinal manipulative therapy (SMT). The RCTs suggest that physiotherapy and SMT might be an effective treatment in the management of CEH, but the results are difficult to evaluate, since only one study included a control group that did not receive treatment. Furthermore, the RCTs mostly included participant with infrequent CEH. Future challenges regarding CEH are substantial both from a diagnostic and management point of view.

## Introduction

Cervicogenic headache (CEH) is a secondary headache characterized by unilateral headache and symptoms and signs of neck involvement [1–5]. It is often worsened by neck movement, sustained awkward head position or external pressure over the upper cervical or occipital region on the symptomatic side [1, 3].

The prevalence of CEH varies in the general population depending on the diagnostic criteria, i.e. 1.0 % applying six positive criteria of the Cervicogenic Headache International Study Group (CHISG) and 4.6 % when only five criteria were used, while it was 2.5 % applying the International Headache Society (IHS) criteria [3, 5–8]. A recent epidemiological survey found that the prevalence was 0.13 % in men and 0.21 % in women applying three or more major CHISG criteria [9, 10]. Thus, along with different diagnostic criteria, it is likely that other methodological differences play a role.

The pathogenesis of CEH may originate from various anatomic structures in the cervical spine. Convergence of afferents of the trigeminal and upper three cervical spinal nerves onto the second-order neurons in the trigemino-cervical nucleus in the upper cervical spinal cord is likely to lead to the headache [11, 12]. The craniovertebral junction is stabilized by joint capsules, tectorial membrane, transverse and alar ligaments [13]. A proton-weighted magnetic resonance imaging (MRI) study of people with CEH, whiplash-associated headache or migraine was analyzed blinded and identified no significant differences in the three groups [14]. Furthermore, the site of the CEH was not correlated with the site of signal intensity changes of the alar and transverse ligaments. One study suggests that lower cervical disc prolapse may cause CEH [15], but the results could not be confirmed as no specific MRI changes of cervical discs or craniovertebral ligaments were found in CEH [14]. Muscle tenderness is likely to play a role and is more pronounced on the pain than the non-pain site, i.e. pericranial tenderness was recorded according to the tenderness score of eight pairs of pericranial muscles and tendon insertion points, each scored on a four-point scale (0–3) at each location, and the tenderness score on the pain and non-pain sides was compared [10]. However, so far the pathogenesis and etiology of CEH remain a challenge.

Due to insufficient pharmacological treatment strategies, medication overuse is frequent and likely secondary to the pain rather than a confounding factor, as the medication overuse is of shorter duration than the duration of the CEH [10]. A 3-year follow-up of people with CEH from the general population found no improvement [16], while people from the general population with headache attributed to chronic rhinosinusitis or medication overuse headache improved after a short advice [16, 17].

Thus, due to muscle tenderness and possibly not yet identified local factor in the cervical spine, it might be that manual therapies can alleviate CEH, along with blockage of the greater occipital nerve (GON), which is the only effective pharmacological treatment so far [18, 19]. This paper systematically review randomized clinical trials (RCT) assessing the efficacy of manual therapies for CEH.

## Methods

The literature search was done on CINHAL, Cochrane, Medline, Ovid and PubMed. Search words were cervicogenic headache and chiropractic, manipulative therapy, massage therapy, osteopathic treatment, physiotherapy or spinal mobilization. All RCT written in English using either of the manual therapies on CEH were evaluated. CEH was preferentially classified according to the criteria of the IHS from 1988 or its revision from 2004, or according to the Cervicogenic Headache International Study Group (CHISG) [1–5]. Table 1 shows the diagnostic criteria for CEH. The studies had to evaluate at least one CEH outcome measure, i.e. pain intensity, frequency, or duration. The methodological quality of the included RCT studies was assessed by the first author. Table 2 shows that the evaluation covers study population, intervention, measurement of effect, data presentation and analysis and the maximum score is 100 points, and ≥50 points is considered to be methodology of good quality [20–23].

**Table 1:** Diagnostic criteria of cervicogenic headache

*Cervicogenic Headache International Study Group* [3]		
Major criteria^a^	1.	Symptoms and signs of neck involvement
		a. Precipitation of head pain, similar to the usually occurring one:
		i. By neck movement and/or sustained awkward head positioning, and/or:
		ii. By external pressure over the upper cervical or occipital region on the symptomatic side
		b. Restriction of range of motion (ROM) in the neck
		c. Ipsilateral neck, shoulder, or arm pain of a rather vague nonradicular nature or, occasionally, arm pain of a radicular nature.
	2.	Confirmatory evidence by diagnostic anesthetic blockade
	3.	Unilaterality of the head pain, without side shift
Head pain characteristics	4.	a. Moderate-severe, non-throbbing, and non-lancinating pain, usually starting in the neck.
		b. Episodes of varying duration
		c. Fluctuating, continuous pain
Other characteristics of some importance	5.	a. Only marginal effect or lack of effect of indomethacin
		b. Only marginal effect or lack of effect of ergotamine and sumatriptan
		c. Female sex
		d. Not infrequent occurrence of head or indirect neck trauma by history, usually of more than only medium trauma
Other features of lesser importance	6.	a. Nausea
		b. Phonophobia and photophobia
		c. Dizziness
		d. Ipsilateral “blurred vision”
		e. Difficulties swallowing
		f. Ipsilateral edema, mostly in the periocular area
*International Classification of Headache Disorders-II* [5]		
A. Pain, referred from a source in the neck and perceived in one or more regions of the head and/or face, fulfilling criteria C and D		
B. Clinical, laboratory and/or imaging evidence of a disorder or lesion within the cervical spine or soft tissues of the neck known to be, or generally accepted as, a valid cause of headache		
C. Evidence that the pain can be attributed to the neck disorder or lesion based on at least one of the following:		
i. Demonstration of clinical signs that implicate a source of pain in the neck		
ii. Abolition of headache following diagnostic blockade of a cervical structure or its nerve supply using placebo- or other adequate controls		
D. Pain resolves within 3 months after successful treatment of the causative disorder or lesion		

^a^It is obligatory that one or more of phenomena 1a-1c are present

**Table 2:** Criteria list of methodological quality assessment of randomized controlled trials (RCTs) [22]

1. Study population (30 points)
a) Description of inclusion and exclusion criteria (1 point). Restriction to a homogeneous study population (1 point)
b) Comparability of relevant baseline characteristics: duration of complaint (1 point), value of outcome measures (1 point), age (1 point), recurrences (1 point), and radiating complaints/associated symptoms (1 point)
c) Description of the randomization procedure (2 points). Randomization procedure which excluded bias, i.e. random numbers table (2 points)
d) Description of dropouts for each group and their reasons (3 points)
e) Loss to follow-up: less than 20 % loss to follow-up (2 points), OR less than 10 % loss to follow-up (4 points)
f) Sample size: greater than 50 subjects in the smallest group after randomization (6 points), OR greater than 100 subjects in the smallest group after randomization (12 points)
2. Interventions (30 points)
g) Correct description of the manual intervention (5 points). All interventions described (5 points)
h) Pragmatic study: comparison with an existing treatment modality (5 points)
i) Co-interventions avoided in the design of the study (5 points)
j) Comparison with a placebo control group (5 points)
k) Mention of the experience of the therapist (5 points)
3. Measurement of effect (30 points)
l) Placebo controlled studies: patients blinded (3 points), blinding evaluated and fully successful (2 points) OR Pragmatic studies: patients fully naive, evaluated and fully successful (3 points), time restriction of no manual treatments for at least 1 year (2 points)
m) Outcome measures: pain assessment (2 points), global measure of improvement (2 points), functional status (2 points), spinal mobility (2 points), medical consumption (2 points)
n) Each blinded outcome measure mentioned under item M earns 2 points
o) Analysis of post-treatment data (3 points), inclusion of a follow-up period longer than 6 months (2 points)
4. Data presentation and analysis (10 points)
p) Intention-to-treat analysis when loss to follow-up is less than 10 % OR intention-to-treat analysis as well as worst-case analysis for missing values when loss to follow-up is greater than 10 % (5 points)
q) Corrected presentation of the data: mean or median with a standard deviation or percentiles for continuous variables (5 points)

## Results

The literature search identified seven RCT on CEH that met our inclusion criteria. One study applied physiotherapy ± temporomadibular mobilization techniques and six studies applied cervical spinal manipulative therapy (SMT) [24–30]. Four studies were conducted by chiropractors, two studies by physiotherapists and one by a physician. RCTs studies on massage therapy, spinal mobilization or osteopathic intervention on CEH were not identified.

### Methodological quality of the RCTs

Table 3 shows the methodological score of the included RCT studies. The score varied from 50 to 81 points.

**Table 3:** Quality score of the analyzed randomized controlled trials (RCTs) using manual therapies for treatment of CEH

Study	A	B	C	D	E	F	G	H	I	J	K	L	M	N	O	P	Q	Total
Piekartz and Lüdtke [30]	2	3	4	3	2	0	10	5	0	0	5	2	6	6	3	0	5	56
Nilsson [24]	2	2	4	3	4	0	10	5	5	0	0	2	4	4	3	0	5	53
Nilsson et al. [25]	2	2	4	3	4	0	10	5	5	0	0	2	4	4	3	0	5	53
Jull et al. [26]	2	5	4	3	4	0	10	5	5	5	5	2	8	8	5	5	5	81
Haas et al. [27]	2	4	4	3	4	0	10	5	0	0	5	2	6	0	3	5	5	58
Borusiak et al. [28]	2	2	4	0	4	0	10	0	5	5	5	2	6	0	0	0	5	50
Haas et al. [29]	2	4	4	3	4	0	10	5	0	5	5	2	6	0	3	5	5	63

The letters correspond with letters from the criteria list (Table 2)

### Randomized controlled trials (RCT)

Table 4 shows details from the seven RCT studies regarding study population, intervention and results in relation to headache frequency, duration and intensity, while other results are presented in the text.

**Table 4:** Randomized controlled trials (RCTs) of physiotherapy and spinal manipulative therapy for CEH

Country	Study population	Method	Intervention	Results
*Physiotherapy*				
The Netherland [30]	43 participants (16M, 27F) Age 18–65 years Mean age 36 years CEH mean duration >6 months and at least one of four signs of temporomandibular disorder (TMD) CEH diagnosed by neurologist	RCT of 7–7½ months duration conducted by a physiotherapist, i.e. Baseline evaluation 3- to 6-week treatment 6-month follow-up Comparison of baseline, post-treatment and at 6-month follow-up	Six interventions ≤30-min within 3–6 weeks The physiotherapist selected the technique and treatment and exercise he or she considered to be beneficial for the participant The experimental group received accessory (translatory) movements of temporomadibular region and/or masticatory muscle techniques such as trigger point treatment and muscle stretching. Active and passive movement facilitating optimal function of cranial nerve tissue, coordination exercises and home exercises. The therapist could also opt for additionally neuromusculoskeletal treatments for cervical region (*n* = 20) (7M, 13F) Conventional physiotherapy including manual techniques at the cranio-cervical region and exercises (*n* = 18) (6M, 12F) Drop outs (*n* = 5)	The experimental group showed a significant reduction in headache intensity 3- and 6-month post-treatment as compared to conventional physiotherapy (*p* < 0.001) The pain intensity was seven on a colored analog scale (comparable to VAS) at baseline and reduced to 3.2 and 2.1 at 3- and 6-month post-treatment in the experimental group, while the pain was stable around 6.8 in the conventional physiotherapy group at the three recordings
*Spinal manipulative therapy (SMT)*				
Denmark [24]	39 participants (17M, 22F) Age 20–57 years Mean 39 years Headache ≥5 days per month for at least 3 months CEH diagnosed by a physician and chiropractor	RCT of 6-week duration conducted by a chiropractor, i.e. 2-week baseline 3-week treatment 1-week follow-up Comparison of pre-treatment at week 2 and post-treatment at week 6 Headache diary	Cervical SMT by chiropractor i.e. toggle recoil at upper cervical and diversified technique at lower cervical determined by the chiropractor (*n* = 20) (9M, 11F) Soft tissue (ST) work including deep friction massage at cervico-thoracic area and laser therapy at upper cervical region (*n* = 18) (8M, 10F) Drop outs (*n* = 1)	Mean headache duration was reduced in both the CSMT and ST group (*p* < 0.0001 and *p* < 0.002, respectively), i.e. a 59 and 52 % reduction from pre- to post-treatment. Mean headache duration reduction was not statistically significant in the two groups Mean headache intensity was reduced in the cervical SMT group (*p* < 0.001), but not in ST group, i.e. a 36 and 22 % reduction from pre- to post-treatment Mean headache intensity reduction was not statistically significant in the two groups
Denmark [25]	54 participants (23M, 31F) Age 20–60 years Mean 37 years Headache ≥5 days per month for at least 3 months CEH diagnosed by physician and chiropractor	RCT of 5-week duration conducted by a chiropractor, i.e. 1-week baseline 3-week treatment 1-week follow-up Comparison of pre-treatmen at week 1 and post-treatment at week 5 Headache diary	Cervical SMT by chiropractor i.e. toggle recoil at upper cervical and diversified technique at lower cervical determined by the chiropractor (*n* = 28) (13M, 15F) Soft tissue (ST) work including deep friction massage at cervico-thoracic area and laser therapy at upper cervical region (*n* = 25) (10M, 15F) Drop outs (*n* = 1)	Median headache duration was reduced in both cervical SMT and ST group (*p* < 0.0001 and *p* < 0.04, respectively), i.e. a 69 and 37 % reduction from pre- to post-treatment Median headache duration was reduced more in the cervical SMT than in the ST group (*p* < 0.03) Median headache intensity was reduced in the cervical SMT group (*p* < 0.0015), but not in ST group, i.e. a 36 and 17 % reduction from pre- to post-treatment. Median headache intensity was reduced more in the cervical SMT than in the ST group (*p* < 0.04)
Australia [26]	200 participants (60M, 140F) Age 18–60 years Mean 36.7 years Mean headache duration 6.1 years CEH diagnosed by GPs or physiotherapists	RCT of 12-month duration conducted by physiotherapists i.e. 2-week baseline 6-week treatment 3-, 6- and 12-month follow-up Comparison of baseline, immediately following post-treatment and 12-month follow-up Headache diary recording	Cervico-scapular muscle exercise twice a day (*n* = 52) (9M, 43F) Cervical SMT a total of 8-12 treatments ≤30-min (*n* = 51) (19M, 32F) Combined cervical SMT and cervico-scapular muscle exercise (*n* = 49) (21M, 28F) Control group (no treatment) (*n* = 48) (11M, 37F) Drop outs (*n* = 7)	Headache frequency and intensity were reduced immediately following post-treatment and at 12-month follow-up in all intervention groups as compared to the control group (*p* < 0.001–0.05) A 50 % reduction in headache frequency was noted in 76 % in cervico-scapular muscle excercise group, 71 %, in the cervical SMT group, 81 % in the combined cervical SMT and exercise group and 29 % of the control group, while 100 % reduction was observed in 31, 33, 42 and 4 % of the four groups Headache duration was reduced in the cervical SMT and the combined cervical SMT and exercise group immediately following post-treatment (*p* < 0.05 and 0.001 respectively) and in the combined cervical SMT and exercise group at 12-month follow-up (*p* < 0.05)
USA [27]	24 participants (4M, 19F, 1 unknown) Mean age 40.3 years Mean headache duration >3 months CEH diagnosed by chiropractor	RCT of 12-week duration conducted by three chiropractors, i.e. baseline evaluation 3-week treatment Follow-up at weeks 4 and 12 Comparison of baseline, 4 (1 week post-treatment) and 12-week follow-up Headache diary recording	All participants received cervical SMT by the diversified technique, with an option for additional two physical modalities, i.e. heat and soft tissue therapy including massage and trigger point therapy. Treating chiropractor could also recommend modifications of daily activities and rehabilitative exercises SMT 1 time per week (*n* = 7) (2M, 5F) SMT 3 times per week (*n* = 8) (2M, 8F) SMT 4 times per week (*n* = 8) (8F) Drop outs (*n* = 1)	At 4-week follow-up headache intensity (see result text for details) was significantly reduced in SMT 4 times a week group as compared to SMT one time a week group, and at 12-week follow-up headache intensity was significantly reduced in both the SMT 3 and 4 times a week groups as compared to the SMT 1 time a week group At 4- and 12-week follow-up the mean headache intensity was reduced 21 and 4 % in the SMT 1 time a week group, 49 and 44 % in the SMT 3 times a week group and 58 and 38 % in the SMT 4 times a week group At 4- and 12-week follow-up the mean headache frequency improved 41, 45, 61 %, and 14, 36, 53 % in the SMT 1, 3 and 4 groups, respectively
Germany [28]	52 participants (21M, 31F) Age 7–15 years Mean age 11.6 years At least headache once a week for at least 6 months CEH diagnosed by physician	Prospective RCT of 4-month duration conducted by a physician, i.e. 2-month baseline One treatment 2-month follow-up Comparison of baseline, post-treatment, follow-up Headache diary recording	Cervical SMT by physician (*n* = 24) Sham cervical manipulation (Placebo) (*n* = 28) Drop outs (*n* = 4)	Both the treatment and sham group had a statistically significant reduction in headache days from baseline to 2-month follow-up (*p* = 0.009 and *p* = 0.027), i.e. from 40.4 to 30.7 % days with headache, and from 41.2 to 31.8 % days with headache Headache frequency, total duration and intensity showed no statistical significant change in neither of the two groups No statistically significant differences were found between the two group in relation to the headache variables described above
USA [29]	80 participants (16M, 64F) Mean age 36 years Mean headache duration >3 months CEH diagnosed by chiropractor	Prospective RCT of 6-month duration conducted by four chiropractors, i.e. Baseline evaluation 8-week treatment Follow-up at weeks 12 and 24 Comparison of baseline, 12- and 24-week follow-up Headache diary recording	All interventions received 10 min visits by chiropractors One cervical and upper thoracic SMT treatment every week, with prior optional 5 min moist heat pack and 2 min light massage and 8 visit including control physical examinations but no treatment (*n* = 20) (4M, 16F) Two cervical and upper thoracic SMT every week, with prior optional 5 min moist heat pack and 2 min light massage (*n* = 20) (4M, 16F) One 5 min of moist heat followed by 5 min light massage every week and eight control physical examination but no treatment (*n* = 20) (5M, 15F) Two 5 min of moist heat followed by 5 min light massage every week (*n* = 20) (3M, 17F) Drop outs (*n* = 7)	Headache intensity at 4, 12 and 24 weeks improved more in the SMT group than in light massage group that received treatment twice a week (*p* < 0.05), while a similar comparison among those whom received treatment once a week was not statistical significant At 24 weeks mean headache intensity was reduced 35 and 45 % in the SMT groups treated once or twice a week, while it was reduced 27 and 17 % in the similar light massage groups At 24 weeks a 50 % reduction in pain intensity was achieved by 28 and 47 % in the SMT groups treated once or twice a week, while it was 28 and 16 % in the similar light massage groups At 24 weeks mean headache frequency was reduced 48 and 56 % in the SMT groups treated once or twice a week, while it was reduced 35 and 31 % in the similar light massage groups

### Physiotherapy

The Dutch study was conducted by experienced physiotherapists with unblinded treatment and outcome measures [30]. The participants were diagnosed with CEH by a neurologist according to the criteria of the International Headache Society (IHS) [5]. Participants were excluded, if ever received temporomandibular disorder (TMD)/orthodontic treatment or experienced neuropathic head pain. The primary end point was headache intensity while TMD complaints (mouth opening, pain and range, deviation, sounds and pain threshold of anterior temporal muscles) and neck disability were secondary end points. Both TMD complaints and neck disability improved statistically significantly in the experimental group as compared to conventional physiotherapy group at 3- and 6-month follow-up (*p* < 0.001 in both comparisons).

### Cervical spinal manipulative therapy

The Danish study was conducted by a chiropractor with unblinded treatment and blinded analysis of outcome measures [24]. The participants were diagnosed by a physician according to the criteria of the IHS excluding the radiographic criterion [1]. Participants whom had not previously received SMT or had conditions contraindicated to SMT were excluded from the study. The primary end-points were change in headache intensity, headache duration and NSAIDs consumption from pre-treatment at 2 weeks to post-treatment at week 6. The consumption of NSAIDs was significantly reduced from pre-treatment to post-treatment in the cervical SMT group (*p* < 0.0005), but not in the soft tissue (ST) group, however, the reduction in consumption of NSAIDs was not statistically significantly different in the cervical SMT and ST groups (*p* = 0.14).

The 2nd Danish study was based on an extended study population from the 1st Danish study [24, 25]. The methodology and end-points were similar, except that the pre-treatment period was reduced from 2 to 1 week and the statistical calculation was based on median rather than mean change. The consumption of NSAIDs was significantly reduced from pre-treatment to post-treatment in the cervical SMT group, but not in the ST group, however, the median reduction in consumption of NSAIDs was not statistically significantly different in the cervical SMT and ST groups (*p* = 0.14).

The Australian multicenter study was conducted by 25 experienced physiotherapists with unblinded treatment and blinded outcome measures [26]. The study participants were diagnosed according to the diagnostic criteria of CHISG by GPs or physiotherapists [4]. Those with bilateral headache, conditions that contraindicated to spinal manipulative treatment (SMT) or whom had received physiotherapy or chiropractic treatment for headache within the previous year were excluded. The primary end-point was a change in headache frequency from baseline to immediately after treatment and 12 months after the intervention, while headache intensity, duration and neck pain were secondary end-points. Neck pain was reduced immediate post-treatment in the all intervention groups (*p* < 0.001–0.01), but was only maintained at 12-month follow-up in the exercise group and combined SMT and exercise group (*p* < 0.001–0.01). The median medication intake comparing baseline with 12-month follow-up was reduced 93 % in the combined SMT and exercise group, 100 % in the SMT and exercise groups, while it increased 33 % in the control (*p* < 0.015 for all). The authors suggest that the treatment effect may be underestimated since 46 % of controls received active intervention (unspecified) for their headache within the trial period.

An American study conducted by three experienced chiropractors evaluated the dose response for chiropractic care of cervicogenic headache [27]. The participants were diagnosed according to the IHS criteria except the radiographic criterion [1]. Participants were excluded if SMT was contraindicated or if the participants had complicated condition that might have been related to clinical outcome. The primary end-point was a change in mean headache intensity from baseline to 4- and 12-week follow-up recorded on 100 points modified Von Korff pain scale. The headache intensity score is the average of headache intensity today, worst headache intensity within the last 4 weeks and average headache intensity within the last 4 weeks. Headache frequency, disability, and neck pain were secondary end-points. Although the participants were allowed to seek consultations outside the trial, only few used that opportunity. The main results of the RCT were that several consultations seem to be advantageous over few consultations in the treatment of cervicogenic headache (Table 2). At 4- and 12-week follow-up headache disability was reduced 44, 50, 76 % and 14, 52, 55 % in the SMT 1, 3 and 4 times a week groups, while neck pain was reduced by 31, 50, 55 % and 30, 54, 38 %, respectively. Comparison of the SMT 1 time a week group with SMT 3 and 4 times a week groups was not statistically significant.

The German study was conducted by a physician with blinded participants and unblended treatment and outcome measures [28]. The study followed the guidelines of the IHS with slight modifications, as the diagnosis CEH according to the criteria can only be given retrospectively after resolution of the symptoms [5]. Participants were allowed to have co-occurrence of migraine and/or tension-type headache. Participants were excluded if ever exposed to SMT or diagnosed with secondary headaches other than CEH. Main outcome measures were headache frequency, duration, intensity, medication consumption and days of absence from school. No statistical significant change was observed in the treatment or sham group between baseline and at 2-month follow-up in relation to medicine consumption or days of absence from school due to headache.

The 2nd American pilot study was conducted by four experienced chiropractors while additional chiropractors in each clinic served as a backup therapist [29]. The treatment and outcome measures were unblinded. Participants were diagnosed according to the IHS excluding the radiographic criteria using a questionnaire [5]. Participants were excluded if they could not attend two visits per week for 8 weeks, took prophylactic prescribed medication for headache, had massage or SMT for their headache within the last 3 months or had complicated conditions. The primary end-point was headache intensity while secondary end-points were headache frequency, disability, neck pain and use of over the counter medication (OTC). At 24 weeks mean neck pain and mean neck disability were reduced 28 and 52 % in the SMT group treated once a week, 47 and 52 % in the SMT group treated twice a week, 29 and 45 % in the light massage (LM) group treated once a week, and 18 and 20 % in the LM group treated twice a week. The authors concluded that only the SMT group treated twice a week had clinical important effect on mean neck pain and disability. Generally dose effects tended to be small.

## Discussion

### Methodological considerations

All seven RCTs studies ascertained the participants through clinical interviews which is considered to be the most valid method in establishing a precise headache diagnosis [31]. All the RCTs included relatively few participants except the Australian physiotherapy study [26]. However, due to participants were divided into four groups each with 48–52 participants, even the Australian study did not receive points for number of participants in the quality score (Table 3). The number of investigators in the seven RCTs varied from 1 to 25. The advantage with one investigator is elimination of inter-observer variability, which is likely to be present if there are two or more investigators. The 25 investigators in the Australian study might be a challenge in relation to the result [32]. The Dutch study was flawed by the participants not being blinded to the intervention, as well as co-intervention was allowed by the investigator which is a major risk for bias [30]. All the RCTs were considered to be of at least good methodological quality, i.e. score ≥50 (Table 3), with the Australian study standing out with an excellent 81 points score of the maximum 100 points.

According to the guidelines of the IHS, an intervention is recommended to last at least 3 months in chronic migraine trials [33]. All the RCTs had less than 3-month intervention, varying from a single treatment to 8-week treatment. In three of SMT the RCTs allowed non-trial treatment which can lead to biased results [26, 27, 29]. Two of the RCTs included participants with co-occurrence of migraine and tension-type headache [28, 29], thus, the effect observed might not be exclusively due to improvement of the CEH.

Only one of the RCTs included a control group that did not receive treatment [26]. It is generally accepted that RCTs including a control group are advantages to pragmatic RCTs, as the effect in the placebo control group often is high [23]. True net effect is more accurately calculated when adding a control group. One RCT had had a successful blinding using SMT or sham treatment, the latter group was denoted as “control group” by the authors [28]. Future RCTs should include a placebo group, i.e. a group of participant that do not receive treatment, although, it is known that blinding adult participants in SMT trials is difficult [34]. Thus, the lack of control group that do not receive treatment makes interpretation of the results difficult, since many of the RCTs had “control groups” that receive a non SMT treatment that might had some effect.

### Results

The Dutch study was considered to be of good methodological quality, although it had room for many improvements [30]. The experimental group had a statistically significant improvement in headache intensity as compared to conventional physiotherapy, an effect that must be considered to also be of clinical significance as the headache intensity was reduced >50 %. The study stands alone, since it also included TMD complaints that also improved. The study included multimodal treatment modalities such as exercise, and thus the results cannot with certainty be exclusive of manual intervention.

The two Danish studies were based on the same study population, with additional 15 participants in the 2nd Danish study [24, 25]. The 1st Danish study presented mean data and the 2nd Danish study presented median data. The median but not mean headache duration and intensity was statistically significantly reduced in the SMT group as compared to the ST group [24, 25]. The 59 and 52 % mean reduction of headache duration in the SMT and ST groups is clinically meaningful, and the 36 and 22 % mean reduction in headache intensity in the two groups is also likely to be clinically meaningful.

The Australian study showed a significant reduction in headache frequency and intensity in all active treatment groups as compared to the control group, an effect that was maintained at 12-month follow-up [26].

The 1st American RCT was a dose–response study without statistical significant results, but there was a tendency toward favouring SMT three or four times a week for SMT once a week [27]. The study did not avoid co-intervention in the any of the three groups leading to a possible bias.

The German RCT included children and adolescent and had only one treatment session, and found no statistically significant differences [28]. Due to the single treatment, it cannot be excluded that more treatment sessions might have given another result, considering that CEH is known to be difficult to treat.

The results of the 2nd American study favoured SMT for light massage (LM), and favouring SMT four times a week slightly over SMT three times a week [29].

One of the major problems in all the RCTs is the fact that the majority of participants had intermittent CEH [24–30]. However, CEH is often characterized by a continuous headache with an intensity that might fluctuate rather than being a paroxysmal disorder [10, 14]. The fact that CEH is often continuous makes sense, assuming that CEH is caused by local factors in the neck/cervical spine. Another major problem is the fact that clinical diagnostic criteria for CEH have not proved to be valid [35]. Although, applying the diagnosis criteria of CHISG not including a blockage of the greater occipital nerve (GON) is equally inter-observer reliable as the diagnosis of migraine and tension-type headache [36]. Thus, the validity of a GON blockage as a diagnostic criteria can be questioned. Medication is usually ineffective in CEH. So far there have not been conducted any RCTs on the effect of medicine in CEH. Blockage of the GON might be effective in CEH [10, 18, 19]. However, an operation of the peripheral course of GON with special attention to the trapezius insertion had no effect [37].

## Conclusion

Current RCTs suggest that physiotherapy and SMT might be an effective treatment in the management of CEH. However, the RCTs mostly included participant with infrequent CEH. Future challenges regarding CEH are substantial both from a diagnostic and management point of view.
